# Deep Learning Algorithm Trained with COVID-19 Pneumonia Also Identifies Immune Checkpoint Inhibitor Therapy-Related Pneumonitis

**DOI:** 10.3390/cancers13040652

**Published:** 2021-02-06

**Authors:** Carlo Augusto Mallio, Andrea Napolitano, Gennaro Castiello, Francesco Maria Giordano, Pasquale D’Alessio, Mario Iozzino, Yipeng Sun, Silvia Angeletti, Marco Russano, Daniele Santini, Giuseppe Tonini, Bruno Beomonte Zobel, Bruno Vincenzi, Carlo Cosimo Quattrocchi

**Affiliations:** 1 Departmental Faculty of Medicine and Surgery, Unit of Diagnostic Imaging and Interventional Radiology, Università Campus Bio-Medico di Roma, 00128 Rome, Italy; g.castiello@unicampus.it (G.C.); f.giordano@unicampus.it (F.M.G.); p.dalessio@unicampus.it (P.D.); b.zobel@unicampus.it (B.B.Z.); c.quattrocchi@unicampus.it (C.C.Q.); 2 Departmental Faculty of Medicine and Surgery, Unit of Medical Oncology, 00128 Rome, Italy; m.russano@unicampus.it (M.R.); D.Santini@unicampus.it (D.S.); g.tonini@unicampus.it (G.T.); b.vincenzi@unicampus.it (B.V.); 3 Department of Interventional Radiology, S. Maria Goretti Hospital, 04100 Latina, Italy; m.iozzino@ausl.latina.it; 4 Infervision Europe GmbH, Mainzer Strasse 75, D-65189 Wiesbaden, Germany; syipeng@infervision.com; 5 Departmental Faculty of Medicine and Surgery, Unit of Clinical Laboratory Science, Università Campus Bio-Medico di Roma, 00128 Rome, Italy; S.Angeletti@unicampus.it

**Keywords:** artificial intelligence, COVID-19, deep learning, chest CT, immune checkpoint inhibitors therapy, drug-induced pneumonitis

## Abstract

**Simple Summary:**

The use of immune checkpoint inhibitors (ICIs) to treat oncologic diseases is progressively increasing. Computed tomography (CT) features of ICI therapy-related pneumonitis may overlap with other diseases, including coronavirus disease 2019 (COVID-19). Thus, oncologic patients undergoing ICI therapy and developing pneumonitis are at risk of being misdiagnosed. Exploring the strengths and weaknesses of artificial intelligence in distinguishing between ICI therapy-related pneumonitis and COVID-19 is of great importance for oncologic patients and for clinicians in order to increase awareness on this topic and stimulate novel strategies aimed to promptly and correctly classify and treat this category of vulnerable patients.

**Abstract:**

Background: Coronavirus disease 2019 (COVID-19) pneumonia and immune checkpoint inhibitor (ICI) therapy-related pneumonitis share common features. The aim of this study was to determine on chest computed tomography (CT) images whether a deep convolutional neural network algorithm is able to solve the challenge of differential diagnosis between COVID-19 pneumonia and ICI therapy-related pneumonitis. Methods: We enrolled three groups: a pneumonia-free group (*n* = 30), a COVID-19 group (*n* = 34), and a group of patients with ICI therapy-related pneumonitis (*n* = 21). Computed tomography images were analyzed with an artificial intelligence (AI) algorithm based on a deep convolutional neural network structure. Statistical analysis included the Mann–Whitney U test (significance threshold at *p* < 0.05) and the receiver operating characteristic curve (ROC curve). Results: The algorithm showed low specificity in distinguishing COVID-19 from ICI therapy-related pneumonitis (sensitivity 97.1%, specificity 14.3%, area under the curve (AUC) = 0.62). ICI therapy-related pneumonitis was identified by the AI when compared to pneumonia-free controls (sensitivity = 85.7%, specificity 100%, AUC = 0.97). Conclusions: The deep learning algorithm is not able to distinguish between COVID-19 pneumonia and ICI therapy-related pneumonitis. Awareness must be increased among clinicians about imaging similarities between COVID-19 and ICI therapy-related pneumonitis. ICI therapy-related pneumonitis can be applied as a challenge population for cross-validation to test the robustness of AI models used to analyze interstitial pneumonias of variable etiology.

## 1. Introduction

SARS-CoV-2 (severe acute respiratory syndrome coronavirus 2) is a novel coronavirus that was first identified in Wuhan, China, and that is responsible for a highly contagious respiratory disease named coronavirus disease 2019 (COVID-19) [1,2]. Due to its rapid global spread, on 11 March 2020, the World Health Organization (WHO) officially characterized COVID-19 as pandemic [3].

Coronaviruses are composed of four major structural proteins, including the envelope (E) protein, the membrane (M) protein, the nucleocapsid (N) protein, and the spike (S) protein, the latter being of paramount importance since it mediates viral attachment to the host cell membrane receptor [4].

Real-time reverse transcription polymerase chain reaction (RT-PCR) and next-generation sequencing methods applied to respiratory tract specimens (e.g., nasopharyngeal or oropharyngeal swab) are considered the reference standard for the diagnosis of SARS-CoV-2 infection [5].

Computed tomography (CT) of the chest can detect lung manifestations, which are often associated with COVID-19 [6,7,8]. The CT features of COVID-19 pneumonia are nonspecific and may overlap with those of other types of pneumonia and pneumonitis [9], including immune checkpoint inhibitor (ICI) therapy-related pneumonitis.

Recently, the use of ICI therapy as first- and second-line treatment of different types of malignancies has rapidly grown [10]. ICIs act through immune system-mediated destruction of tumor cells [11,12]. ICI therapy-related pneumonitis [13,14] is an uncommon but important immune-related adverse event, with potential significant morbidity and mortality. Importantly, given the expanding population of cancer patients exposed to ICIs, the number of therapy-related pneumonitis is expected to escalate in the near future [15,16]. As the clinical manifestation is often nonspecific, CT plays a critical role in the diagnosis of ICI therapy-related pneumonitis.

Interestingly, COVID-19 pneumonia and ICI therapy-related pneumonitis have been suggested to share critical biological mechanisms, including the hyperactivation of immune cells associated with a significant increase in proinflammatory cytokines [17]. Indeed, for both COVID-19 pneumonia [18] and ICI therapy pneumonitis [19], the use of tocilizumab, a recombinant humanized monoclonal antibody inhibiting the human IL-6 receptor, originally developed for the treatment of rheumatoid arthritis, is under scrutiny [20].

Distinguishing between COVID-19 pneumonia and ICI therapy-related pneumonitis is a diagnostic challenge.

Artificial intelligence (AI), using deep learning technology, is highly promising in the medical imaging field due to its capability of feature extraction and analysis [9,21,22]. It has been also applied to detect various imaging features of chest CT [23,24], allowing qualitative and quantitative analysis, which could provide an estimation of the disease burden, facilitating and expediting imaging interpretation [25,26].

In the present study, given the similar biological and clinical characteristics of COVID-19 pneumonia and ICI therapy-related pneumonitis, we tested whether a deep learning algorithm is able to distinguish between COVID-19 pneumonia and ICI therapy-related pneumonitis.

## 2. Materials and Methods

We designed a retrospective observational study. This study was performed in accordance with the Declaration of Helsinki. The local Ethical Committee approved the study (the ethic code is: Prot.: 88/20 OSS.NOT ComEt CBM) and waived the written informed consent for the participants.

### 2.1. Participants

All of the subjects underwent chest CT scan and were consecutively sampled from our electronic database. In this study, three groups of patients were included and classified according to both medical history and CT imaging findings (i.e., radiological reports). Inclusion criteria for group selection were as it follows:

COVID-19: a group of consecutive symptomatic (fever > 37.5 °C, dyspnea and/or cough and/or fatigue) patients, with confirmed COVID-19 pneumonia by positive RT-PCR (RealTiMe SARS-CoV-2 Assay, Abbott Laboratories. Abbott Park, IL, USA) on nasopharyngeal (or oropharyngeal) specimen with the swab technique and positive chest CT scans acquired between 15 March and 5 April 2020.

ICI therapy-related pneumonitis: a group of consecutive oncological patients with a positive history of ICI therapy-related pneumonitis and a positive chest CT scan acquired between 2017 and 2019, before the occurrence of any proven case of COVID-19 in Italy. The diagnosis of ICI therapy-related pneumonitis was clinically established based on the absence of other proven microbiological or pharmacological causes of pneumonia, full recovery after drug discontinuation, and medical therapy (mainly based on corticosteroids), and was confirmed by resolution of the findings at follow-up chest CT scan.

Pneumonia-free patients (control group): a group of consecutive symptomatic (fever > 37.5 °C, dyspnea, and/or cough and/or fatigue) patients with a negative chest CT scan acquired between 15 March and 5 April 2020.

### 2.2. Chest CT Imaging Protocol

All chest CT acquisitions were obtained by maintaining the patients in the supine position during end-inspiration, with or without contrast medium injection. Chest CT images were acquired on a Dual Source 384-slice (2 × 192) CT (Siemens SOMATOM Force, Erlangen, Germany (tube real-time voltage modulation: 70–150 kV; tube real-time dose modulation (CARE Dose4D™) 80–250 mAs; spiral pitch factor: 1.8; collimation width: 0.6 mm)), on a 128-slice CT (Siemens SOMATOM Definition AS, Erlangen, Germany (tube voltage: 120 kV; tube real-time dose modulation (CARE Dose4D™) 80–250 mAs; spiral pitch factor: 1.2; collimation width: 0.6 mm)), and on a 40-slice CT (Philips Brilliance CT (tube voltage: 120 kV; 80–150 mAs; spiral pitch factor: 1.0; collimation width: 0.625 mm)).

A meticulous decontamination of the CT room and passive air exchange was conducted after every scan performed on patients with clinical or imaging suspicion of COVID-19.

### 2.3. Artificial Intelligence Analysis

**Figure 1:**
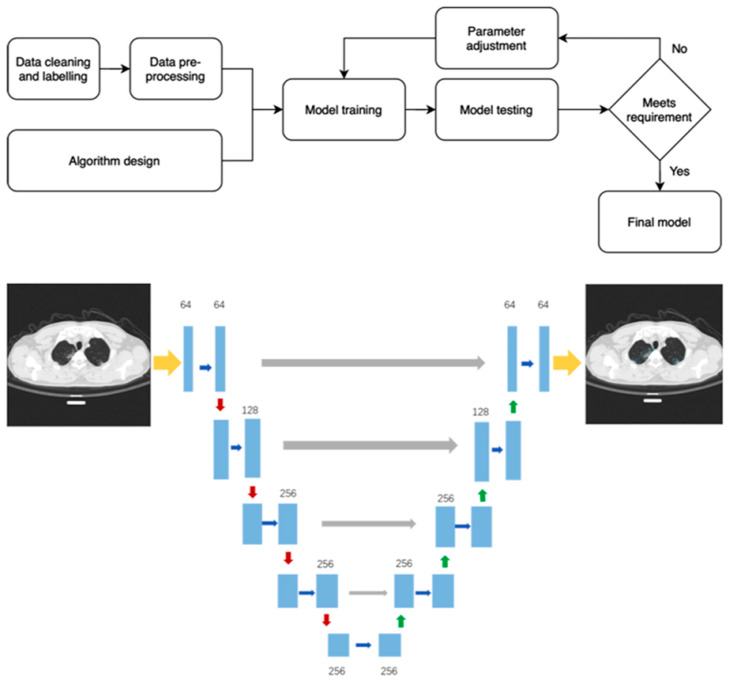
Flowchart of the model training process and a diagram to show how the artificial intelligence (AI) model works to generate a prediction risk of disease.

The artificial intelligence analysis was performed by means of InferRead^TM^ CT Lung (COVID-19) (Infervision, Europe GmbH, Wiesbaden, Germany), an AI solution specifically developed for diagnosis and management support of COVID-19 pneumonia. Among its features, the algorithm module includes automated segmentation of the core features of COVID-19 lung lesions and the segmentation of the lung lobes (right upper lobe, middle lobe, right lower lobe, left upper lobe, left lower lobe). The output also includes the estimated risk probability for the diagnosis of COVID-19 pneumonia. The core algorithm is based on a deep convolutional neural network structure and uses the U-net network structure as the core segmentation network [27]. The model training process is shown in Figure 1. The cleaned and labeled data are trained through the designated network structure. Continuous testing and parameter adjustments allow for the acquisition of a final model that meets the requirements. The model was developed initially after training on a population of patients diagnosed in Wuhan, China, and was later further developed by training on a larger population. Specifically, for the trained AI model, patients’ characteristics (*n* = 2191 adult patients; Wuhan Chinese COVID-19) were mixed, including all stages and clinical presentation of the disease (e.g., symptoms could have been mild, moderate, or severe) [27]. In the Chinese training datasets, controls were 1000 adult patients without COVID-19, who were admitted to Tongji Hospital and had double negative RT-PCR test results. In this group, subjects might or might not have had positive CT findings [27]. The quantitative CT image-derived lesion features analyzed by deep learning model were lung lesion burden volume in terms of cm^3^ or percentages based on automatic opacity segmentation [27].

### 2.4. Statistical Analysis

Descriptive statistics, including means, medians, ranges, and percentiles, were calculated to understand central tendencies of the enrolled cohorts. Data distribution normality was checked by means of Kolmogorov–Smirnov test. The Kruskal–Wallis and the chi-square test were used to compare age and sex distribution among groups, respectively.

This study has three objectives: (1) to assess the AI performance in identifying COVID-19 pneumonia in a population of Italian patients; (2) to test whether the AI could differentiate COVID-19 pneumonia from ICI therapy-related pneumonitis; (3) to test whether the AI could potentially be geared towards the identification and quantitative evaluation of ICI therapy-related pneumonitis.

To investigate the three objectives, we performed the following comparisons: COVID-19 pneumonia vs. pneumonia-free groups, COVID-19 pneumonia vs. ICI therapy-related pneumonitis groups, and ICI therapy-related pneumonitis vs. pneumonia-free groups. COVID-19 disease risk as well as affected lobe percentages and volumes were compared between pairs of groups by using the Man– Whitney U test with a significance threshold of *p* < 0.05. Statistical Package for the Social Sciences (SPSS) software version 26.0 (IBM, Segrate, Milan, Italy) was applied for all the aforementioned statistical computations. Additionally, receiver operating characteristic curve (ROC curve) fitting was performed by using the maximum likelihood fit of a binormal model, and the area under the curve (AUC) was calculated with a 95% confidence interval (95% CI). Sensitivity, specificity, the positive predictive value, and the negative predictive value are presented as point estimates (95% CI).

## 3. Results

### 3.1. Study Population Characteristics

Table 1 shows the study population characteristics and the three independent datasets, namely, the pneumonia-free patients (*n* = 30), patients with COVID-19 pneumonia (*n* = 34), and patients with ICI therapy-related pneumonitis (*n* = 21), used to test the AI model.

The number of males was higher than females across all patient groups (pneumonia-free group: 57%; COVID-19 group: 56%; ICI therapy-related pneumonitis: 67%; *p* = 0.70). Patients with COVID-19 pneumonia and ICI therapy-related pneumonitis were older than pneumonia-free patients (*p* = 0.013). The positive SARS-CoV-2 RT-PCR was available for all patients with COVID-19 and a negative RT-PCR test was available for 14/30 (47%) pneumonia-free patients. Within the ICI therapy-related pneumonitis group of patients, non–small-cell lung cancer (NSCLC) was the most prevalent primary tumor (17/21; 81%). The diagnosis of ICI therapy-related pneumonitis occurred as a complication during immunotherapy with nivolumab (7/21; 33%), pembrolizumab (12/21; 57%), and atezolizumab (2/21; 10%). Three patients (3/21; 14%) received concomitant systemic therapy, with paclitaxel, lenvatinib or carboplatin–etoposide, respectively. Six patients (6/21; 29%) were also under long-term prednisolone in doses of 5 mg/day.

### 3.2. AI Model Performance

The AI processing time for one CT exam was around 10–20 s in a dedicated server with the following configuration characteristics: 16GB RAM, 3TB Drive, GPU-powered Linux server system. The chest CT studies can be automatically forwarded to the AI server located on premises. Once the server receives any study, the AI application starts to process and store the results of AI until the physicians view them. Two series can be analyzed in parallel given the number of GPU instances available. The vendor agnostic AI system is capable of analyzing the CT images generated by different CT machine vendors. The system is able to accept CT images generated by CT machines in different reconstruction protocols with a reconstruction slice thickness lower than 1.5 mm. The result can be also accessed with a URL to the case worklist. An instant alert is notified on the case worklist page once the chest CT arrives in the AI server and is deemed as COVID-19 suspicious by the AI application.

The performance of the AI model in terms of risk estimation of COVID-19 pneumonia on chest CT images is summarized in Table 2. When testing the COVID-19 pneumonia vs. pneumonia-free groups, the sensitivity for COVID-19 detection was 97.1% (95% CI: 88.6%, 97.1%), and the specificity was 100% (95% CI: 90.4%, 100%). Differently, when analyzing the COVID-19 pneumonia vs. ICI therapy-related pneumonitis groups, the sensitivity for COVID-19 detection was 97.1% (95% CI: 90.9%, 99.8%), and the specificity was 14.3% (95% CI: 4.3%, 18.8%). Finally, when comparing the ICI therapy-related pneumonitis vs. pneumonia-free groups, the sensitivity for COVID-19 detection was 85.7% (95% CI: 71.3%, 85.7%) with a specificity of 100% (95% CI: 83.2%, 100%).

**Figure 2:**
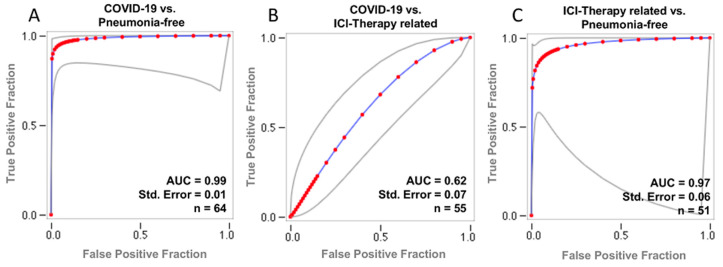
Receiving operating curves (ROC) of the diagnostic performance of the AI prediction risk of COVID-19 pneumonia. Each plot shows the ROC obtained during testing after including the pairs: COVID-19 and pneumonia-free patients (**A**), COVID-19 and ICI-related therapy (**B**) and ICI-related therapy, and pneumonia-free patients (**C**). Gray lines plot 95% confidence intervals. COVID-19 = coronavirus disease 2019. ICI = immune checkpoint inhibitor. AUC = area under ROC. Std. Error = standard error.

The ROC curves for the AI risk prediction of COVID-19 pneumonia are shown in Figure 2. The corresponding AUC values for COVID-19 detection depending on the testing dataset are 0.99 (95% CI: 0.98, 1.00) for patients with COVID-19 pneumonia vs. pneumonia-free patients, 0.62 (95% CI: 0.55, 0.70) for patients with COVID-19 pneumonia vs. patients with ICI therapy-related pneumonitis, and 0.97 (95% CI: 0.91, 1.00) for patients with ICI therapy-related pneumonitis vs. pneumonia-free patients, respectively.

The results of the comparison between COVID-19 pneumonia and ICI therapy-related pneumonitis, in terms of total and lobar involvement, are summarized in Table 3. Considering both the affected lobe percentages and volumes, the left lower lobe appears to be significantly more affected in the COVID-19 group than the ICI therapy-related pneumonitis group (*p* < 0.01).

**Figure 3:**
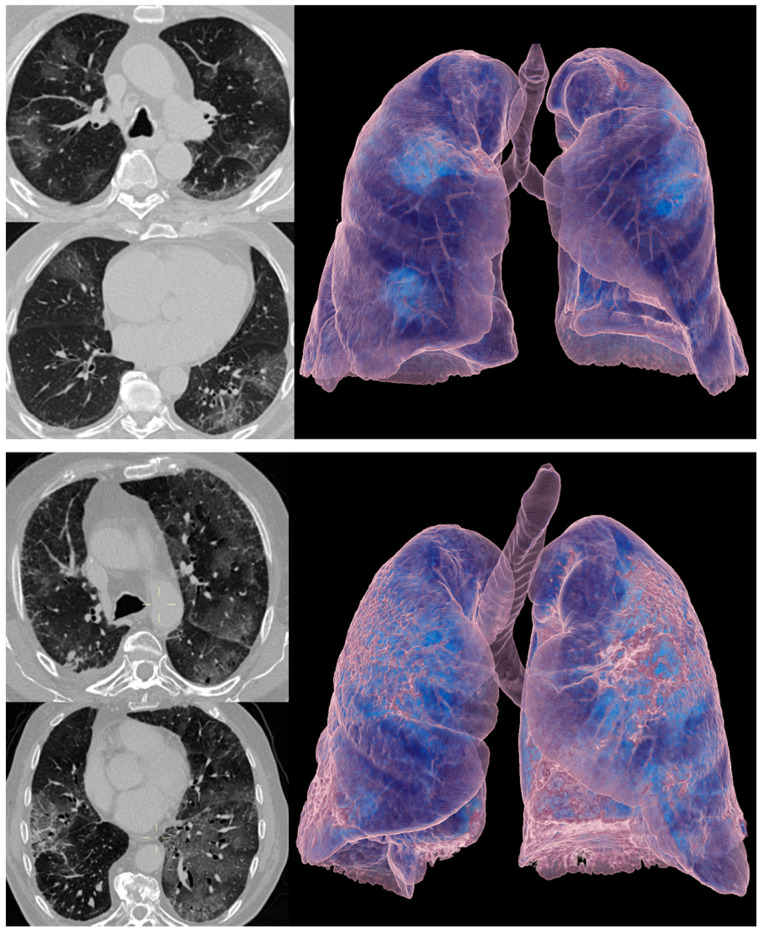
Computed tomography (CT) images of patients with RT-PCR-proven COVID-19 pneumonia (upper panel) and ICI therapy-related pneumonitis (lower panel). Axial CT images with lung window (left side of upper and lower panel) and 3D volume rendering technique (VRT) images (right side of upper and lower panel) are shown. Note the overlapping CT features of ground-glass opacities between the two cases of different etiology.

Representative CT images are shown in Figure 3.

## 4. Discussion

In this study, we assessed the performance of a deep learning algorithm, initially trained on a population of Wuhan in China, in solving the challenge of differential diagnosis between COVID-19 pneumonia and ICI therapy-related pneumonitis.

The patients with RT-PCR confirmed diagnosis of COVID-19 pneumonia, showing the typical CT findings reported in several studies [6,7,28,29,30,31]. The most relevant CT features were multifocal and peripheral (and often bilateral) ground-glass areas associated with subsegmental patchy consolidations, with predominant involvement of the lower lung lobes. As expected, we found excellent accuracy of the algorithm in the detection of COVID-19 pneumonia when compared with pneumonia-free patients (AUC = 0.99). These results support the use of the algorithm as a valid tool for diagnosis and management support of COVID-19 pneumonia in the Italian population, similarly to what was reported in the Chinese population [9], especially in those settings where the automated analysis could be used to rapidly screen patients admitted with symptoms of suspect COVID-19 pneumonia. In this context, based also on the pathological autoptic findings, it should be underlined that the known coexistence of viral and bacterial infections in COVID-19 patients hinders the binary approach used by AI algorithms in the classification of bacterial vs. viral pneumonia or COVID-19 vs. non COVID-19 pneumonia. In real clinical settings, lung consolidations can be the results of more complex clinical patterns, especially in the later stages of disease [32].

ICI therapy-related pneumonitis is an uncommon but important complication of ICI therapy. Since the clinical manifestations of ICI therapy-related pneumonitis are nonspecific, CT plays an important role in the diagnosis of this adverse event. ICI therapy-related pneumonitis might present with several patterns that include organizing pneumonia, nonspecific interstitial pneumonia, and acute interstitial pneumonia–acute respiratory distress syndrome [10]. These patterns seemingly overlap the CT features of COVID-19 pneumonia possibly due to overlapped biological mechanisms. Here, we indeed show that ICI therapy-related pneumonitis was classified by the AI algorithm as COVID-19 pneumonia (AUC = 0.62). These results emphasize the exceptional diagnostic challenge represented by interstitial lung disease in patients with cancer who undergo ICI therapy during the epidemic of COVID-19. Surprisingly, the analysis of lobar involvement showed that the left lower lobe was significantly more affected in COVID-19 patients compared to those with ICI therapy-related pneumonitis. Since ICI therapy-related pneumonitis has been reported to frequently affect the lower lobes [33], the result of the left lower lobe reported in the present study might be influenced by the small sample size. Nevertheless, it should be further investigated in larger cohorts, as it might hint to biological and pathogenic peculiarities of COVID-19 pneumonia with respect to ICI therapy-related pneumonitis.

Given the capability of the COVID-19 algorithm to capture features shared between COVID-19 pneumonia and ICI therapy-related pneumonitis, we were able to show an excellent accuracy of the AI in distinguishing ICI therapy-related pneumonitis from pneumonia-free cases (AUC = 0.97). The result suggests that the algorithm might be geared towards the identification and quantitative evaluation of lungs affected by ICI therapy-related pneumonitis. This interesting hypothesis needs to be further verified with a larger CT dataset of ICI therapy-related pneumonitis, also given the limited sample size of the present study. Additionally, a “transfer learning” strategy, including some ICI patients’ CT data, should be tested and adapted for the partial training of algorithms. This is particularly important, as COVID-19 pneumonia cases are expected to decrease in the future and might become epidemiologically less relevant with the development and distribution of adequate vaccination therapies, whereas the number of ICI therapy-related pneumonitis is predicted to increase according to the increasing usage of ICI in cancer patients [34].

This study has several limitations, including the small size of the samples and the variability of protocols used with the three different scanners. Other viral or bacterial pneumonias were not included in this study; thus, we cannot estimate the accuracy of the AI algorithm for the differential diagnosis with other infectious pneumonias. Moreover, the overlapping chest CT features of several diseases reflect common mechanisms of response of the lungs to different etiologies; therefore, measurements of volume, shape, or density of pulmonary lesions may not be sufficient features to develop powerful deep learning models. Lesion distribution and other radiomics-based data, including clinical information, are promising to further improve the performance of AI algorithms [35].

## 5. Conclusions

In conclusion, the AI model, initially developed on the population of Wuhan, classified ICI therapy-related pneumonitis as COVID-19 pneumonias due to shared features between the two conditions, and it distinguished ICI therapy-related pneumonitis from pneumonia-free controls. Awareness must be increased among clinicians about imaging similarities between COVID-19 and ICI therapy-related pneumonitis.

ICI therapy-related pneumonitis can be applied as a challenge population for cross-validation to test the robustness of the AI models used to analyze interstitial pneumonias of variable etiology.

## Figures and Tables

**Table 1:** Demographic characteristics of the experimental groups. Age reported as median value (minimum and maximum). COVID-19 = coronavirus disease 2019; ICI = immune checkpoint inhibitor; SARS-CoV-2 (severe acute respiratory syndrome coronavirus 2); RT-PCR = reverse transcriptase polymerase chain reaction. NSCLC = non–small-cell lung cancer; ccRCC = clear cell renal cell carcinoma; HCC = hepatocarcinoma; SCLC = small cell lung cancer.

Property	COVID-19	ICI Therapy-Related	Pneumonia-Free
Patients (*n*)	34	21	30
Female/male (*n*)	15/19	7/14	13/17
Age (years)	67 (38–87)	72 (46–82)	59 (32–88)
SARS-CoV-2 RT-PCR (positive/negative/n.a.)	34/0/0	0/0/21	0/14/16
Primary cancer	n/a	NSCLC (*n* = 17)	n/a
		ccRCC (*n* = 1)	
		Breast cancer (*n* = 1)	
		HCC (*n* = 1)	
		SCLC (*n* = 1)	
Immunotherapy drug	n/a	Nivolumab (*n* = 7)	n/a
		Pembrolizumab (*n* = 12)	
		Atezolizumab (*n* = 2)	

**Table 2:** Diagnostic performance of deep learning algorithm across the different group comparisons. Values in parentheses are 95% CI. PPV = positive predictive value, NPV = negative predictive value; Acc = accuracy; AUC = area under the receiving operating curve (ROC). COVID-19 = coronavirus disease 2019. ICI = immune checkpoint inhibitor.

Group	Sensitivity %	Specificity %	PPV %	NPV %	Acc %	AUC
COVID-19 vs. pneumonia-free	97.1 (88.6, 97.1)	100 (90.4, 100)	100 (91.2, 100)	96.8 (87.5, 96.8)	98.4	0.99 (0.98, 1.00)
COVID-19 vs. ICI therapy-related	97.1 (90.9, 99.8)	14.3 (4.3, 18.8)	64.7 (60.6, 66.6)	75.0 (22.6, 98.7)	60.0	0.62 (0.55, 0.70)
ICI therapy-related vs. pneumonia-free	85.7 (71.3, 85.7)	100 (89.9, 100)	100 (83.2, 100)	90.9 (81.7, 90.9)	94.1	0.97 (0.91, 1.00)

**Table 3:** Comparison between COVID-19 pneumonia and ICI therapy-related pneumonia based on total and lobar involvement. Volumes are reported as median values of the relative percentage of lobar involvement and absolute volumes. Values in parentheses are 25% and 75% percentiles that were used instead of minimum and maximum, as the value of 0 was frequent in the distribution. ICI = immune checkpoint inhibitor, COVID-19 = coronavirus disease 2019. RUL = right upper lobe, ML = middle lobe, RLL = right lower lobe, LUL = left upper lobe; LLL, left lower lobe.

ICI Therapy		COVID-19	ICI Therapy-Related	*p*-Value
TOTAL	(%)	2.95 (1.22–8.89)	1.68 (0.28–9.62)	0.27
	(cm^3^)	105.54 (44.68–257.07)	52.03 (6.95–225.0)	0.14
RUL	(%)	0.78 (0.15–5.12)	0.17 (0–10.84)	0.43
	(cm^3^)	7.3 (1.21–31.42)	1.92 (0–14.22)	0.17
ML	(%)	0.24 (0–3.89)	0.28 (0–3.47)	0.66
	(cm^3^)	1.01 (0–7.92)	1.00 (0–13.58)	0.61
RLL	(%)	3.54 (1.19–11.06)	1.48 (0.02–11.80)	0.09
	(cm^3^)	27.14 (8.20–83.30)	4.83 (0–41.02)	0.05
LUL	(%)	0.73 (0.05–5.70)	0 (0–2.81)	0.10
	(cm^3^)	7.22 (0.84–54.28)	0 (0–15.42)	0.04
LLL	(%)	3.99 (0.46–17.56)	0 (0–2.10)	<0.01
	(cm^3^)	16.35 (3.66–85.61)	0 (0–10.97)	<0.01
COVID-19 RISK (%)		41.85 (34.52–51.12)	34.4 (27.2–46.4)	0.16

## Data Availability

Data are available upon request to the corresponding author.

## Abbreviations

SARS-CoV-2	severe acute respiratory syndrome coronavirus 2
COVID-19	coronavirus disease 2019
WHO	World Health Organization
RT-PCR	real-time reverse transcription polymerase chain reaction
CT	computed tomography
ICI	immune checkpoint inhibitors
AI	artificial intelligence
ROC	receiver operating characteristic
AUC	area under the curve
SPSS	Statistical Package for the Social Sciences
